# The Effect of Multiwalled Carbon Nanotubes on Hepatotoxicity of Cd^2+^ in Accumulated Cadmium-Metallothione in Mice

**DOI:** 10.1155/2014/463161

**Published:** 2014-09-02

**Authors:** Qi Wei, Bi Juanjuan, Tian Longlong, Li Zhan, Liu Peng, Wu Wangsuo

**Affiliations:** ^1^Radiochemical Laboratory, Lanzhou University, Lanzhou 730000, China; ^2^Lanzhou Institute of Chemistry Physics, Chinese Academy of Science, Lanzhou 730000, China

## Abstract

The effects of oxidized multiwalled carbon nanotubes (oMWCNTs) on the behavior and hepatotoxicity of Cd^2+^ in accumulated cadmium-metallothionein mice were investigated. The results indicated that, after exposure of oMWCNTs to normal mice, oMWCNTs could not induce the liver to produce metallothionein (MT). When exposing Cd-MT mouse to different doses of oMWCNTs oMWCNTs could cause Cd^2+^ release from the accumulated Cd-MT; subsequently, one part of the free Cd^2+^ was eliminated with blood circulation; the other part adsorbed by oMWCNTs would remain in the tissues together with oMWCNTs. The results of the activities changes of alanine aminotransferase (ALT), aspartate aminotransferase (AST), total bilirubin (TB), and blood urea nitrogen (BUN) in plasma showed that the hepatotoxicity of coexposure was lower than that of single exposure, and the hepatotoxicity and accumulation of oMWCNTs in livers depended strongly on the exposure dosage of oMWCNTs. The histology of liver and kidney tissue also confirmed the previous results. Therefore, the author inferred that MT could be connected with oMWCNTs to reduce their hepatotoxicity, but the detailed mechanism needs to be further studied.

## 1. Introduction

Cadmium (Cd) is an environmental and industrial pollutant that poses a serious health risk to humans and animals. Cd^2+^ has multiple cytotoxic and metabolic effects, such as interfering with the normal actions of essential metals [1], including oxidative stress and altering the activities of various enzymes [2–4]. When Cd is exposed to animals, Cd is distributed mainly to the liver and kidney and accumulated with metallothionein (MT). MT is a small metal-binding protein that is characterized by its high thiol content; 20 of its 61 amino acids are cysteine, all of which are in the reduced state and are involved in its metal-binding properties. At the same time, it was also reported that there were two major isoforms of MT in mammals, designated MT-I and MT-II, which were coordinately regulated in the mouse and the proteins were thought to be functionally equivalent. MT can sequester Cd^2+^ from molecular targets by binding to Cd^2+^ with high affinity and, thus, making it less available for excretion; some researches indicate that MT appears to accumulate Cd in a less toxic form until reaching the critical level after exposure to Cd at nonacute toxic doses [5–9]. Therefore, MT can restrain effectively the toxicity of Cd* in vivo*.

However, the physiological environment is a very complex system. Under certain conditions, Cd^2+^ can be released from its bond with MT in some tissues such as liver, in which toxicity may appear again [10]. As well known, nanomaterials and nanotechnology are developing rapidly in recent years and are paid close attention to in medicine, pharmacy, genetic engineering, and so on, and also there are a lot of researches on the environmental toxicity about them [11–14]. Through oral, intraperitoneal, intravenous, and intragastric exposure ways, researchers study fully the biodistribution, excretion, and toxicity of carbon nanotubes* in vivo* by using isotope tracer technique or fluorescence imaging and find that carbon nanotubes are mainly in the liver, spleen, and lung tissues with a long retention [15–20]; at the same time, due to the unique scale effect and large specific surface area, carbon nanotubes show strong adsorption capacities for metal ions in water system [21]. Therefore, Cd^2+^ should be easy into the living things with nanomaterials in real environment, and that may aggravate toxicity of single carbon nanotubes or Cd^2+^; meanwhile nanomaterials may change the toxicity and metabolic behavior of heavy metal ions* in vivo*; thereby, it was important for human health to study the cotoxicity of carbon nanotubes and heavy metal ions in the organism.

In the present work, the effects of oxidation multiwalled carbon nanotubes (oMWCNTs) on the behavior and hepatotoxicity of cadmium (Cd) were investigated. Because the hormone levels (such as ALT, AST, BUN, and TB) in blood would change when there was some damage in liver or kidney tissues [22–24], the levels of ALT, AST, BUN, and TB in serum/or plasma were also measured to determine the toxicity of Cd^2+^ or oMWCNTs in livers or kidneys.

## 2. Materials and Methods 

### 2.1. Preparation of Oxidized MWCNTs

**Figure 1:**
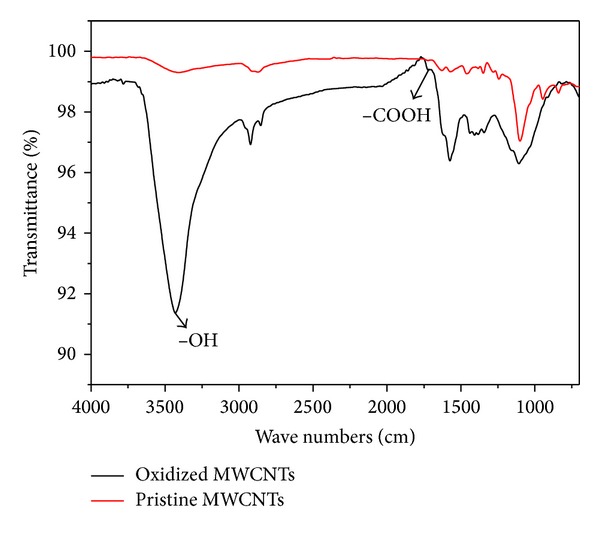
The FT-IRs of oxidized MWCNTs and pristine MWCNTs. The absorption peak of 3400/cm was OH– and that of 1720–1730/cm was COOH–.

**Figure 2:**
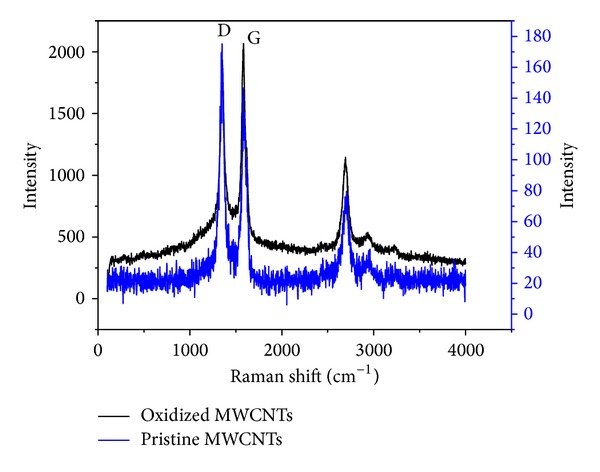
The Raman spectroscopy of oMWCNTs. The D and G band of MWCNT still existed after oxidation, indicating that carbon nanotube structure was not changed.

Multiwalled carbon nanotubes (MWCNTs) were purchased from Shenzhen Nanotech Port Co. Ltd., Guangdong, China, and prepared using chemical vaporization deposition method. According to the product specification, as-received MWCNTs were determined with transmission electron microscopy (TEM) to be 1 to 2 *μ*m in length, with a diameter of 10 to 30 nm. Purity was >96% (wt. %), containing <3% amorphous carbon and <0.2% ash. The as-grown MWCNTs (named as untreated MWCNTs) were added into the solution of 3 mol/L HNO_3_ to remove the metal hemispherical caps on the nanotubes (such as Fe, Ni) that was a byproduct of the synthetic procedure. The mixture of 3 g MWCNTs and 400-mL 3 mol/L HNO_3_ was ultrasonically stirred for 24 h. The suspension was filtrated and rinsed with deionized water until the pH of the suspension reached about 6 and then dried at 80°C. The treated MWCNTs (named oMWCNTs) were calcined at 450°C for 24 h to remove the amorphous carbon [25]. And then oMWCNTs were made FT-IRs and Raman spectroscopy characterization; the results are shown in Figures 1 and 2, respectively. Other chemical reagents, such as HNO_3_, alcohol, and chloroform (ultrapure grade), were purchased from Tianjin Fuyu Chemical Co., Ltd., China; TRIS (ultrapure grade), from Sigma; Sephadex G-75 medium, from Pharmacia 17-0050-02; AST/ALT/BUN/TB (ELISA), from Shanghai Hengdailao Trade Co., Ltd., China.

### 2.2. Animals and Animal Model

Kunming mice (female : male = 1 : 1) initially weighing 15 to 18 g were provided by Laboratory Center for Medical Science, Lanzhou University, Gansu, China. All animals were housed in individual cages in a temperature (21°C to 22°C) and light (from 08:00 to 20:00 hours) controlled environment and were fed food and tap water* ad libitum*. All animal protocols were in accordance with the European Communities Council Directive of November 24, 1986 (86/609/EEC), and approved by Institutional Animal Care and Use Committees of Gansu Province Medical Animal Center and Lanzhou University Animal Committees Guideline. Mice were pretreated with CdCl_2_ (1 mg Cd/kg once daily subcutaneously for 6 days) to accumulate Cd-MT and those mice were referred to as Cd-MT accumulated mice.

### 2.3. The Effects of oMWCNTs on Inducing of Cd-MT

The livers were harvested at 4 h after intravenous exposure of oMWCNTs (about 500 *μ*g/mouse) to Cd-MT model (4 mice) and normal mice (4 mice), respectively. And the content changes curve of Cd-MT and Cd^2+^ in liver was measured, according to the following steps to extract Cd-MT [26]. 4 g of liver tissue was cut into pieces, which were then put into 5 mL of precooling mixed liquid (Volume (Tris-HCl, pH8.2): Volume (CH_3_CH_2_OH): Volume (CH_3_Cl) = 1 : 1.03 : 0.08), homogenate. After adding 20 mL of the mixed liquid into the liver homogenate and mixing them, the liver homogenate was centrifuged for 20 min at 4000 ×g in 4°C; the supernatant was heated at 80°C for 5 min, after cooling in cold water, and centrifuged for 20 min at 4000 ×g in 4°C; and the supernatant was diluted to 4 times volume with −20°C precooled ethanol, stayed overnight at −20°C, and then centrifuged for 30 min at 4000 ×g in 4°C; the precipitation was dissolved fully in 0.01 mol/L Tris-HCl buffer solution and then centrifuged for 20 min at 4000 ×g in 4°C. The supernatant was applied to a Sephadex G-75 column (2 cm × 80 cm) equilibrated with 0.01 mol/L Tris-HCl (pH8.2); the column was eluted with the same buffer solution, and 4 mL fractions were collected to measure the absorbance by Ultraviolet Absorption Spectrophotometry (Lambda 35, PerkinElmer) and the Cd content of fractions was determined through Atomic Absorption Spectrophotometry (A Analyst 700, PerkinElmer).

### 2.4. The Effect of oMWCNTs on Hepatotoxicity of Cd

Three groups of Cd-MT model mice and normal mice (6 mice/group) were exposed to oMWCNTs about 100 *μ*g/mouse, 500 *μ*g/mouse, and 750 *μ*g/mouse, respectively. After 4 h or 24 h, the blood (1 mL), liver, and kidney were harvested and the blood with anticoagulant was used to measure the activities of alanine aminotransferase (ALT), aspartate aminotransferase (AST), total bilirubin (TB), and blood urea nitrogen (BUN) by ELISA testing. The liver, kidney, and blood were digested with the mixture of perchloric acid and 30% of hydrogen peroxide (3 : 1, volume) [27], and then the content of Cd was determined through Atomic Absorption Spectrophotometry. At the same time, the kidney and liver of all groups were obtained and fixed in 10% buffered formalin and processed for routine histology with hematoxylin and eosin stain by the Center for Medical Science, Lanzhou University (Lanzhou, China). Microscopic observation of tissues was carried out with an Olympus Microphot-CX41 microscope coupled with a digital camera.

### 2.5. Statistical Analysis

Data were analyzed for significance using Student's* t-test* and a two-tailed analysis of variance (ANOVA). Differences were considered significant at *P* < 0.05.

## 3. Results

### 3.1. FT-IRs and Raman Spectrum of oMWCNTs

From Figure 1 it could be seen that the surface of carbon nanotubes was combined with a number of –COOH and –OH after oxidization; Figure 2 indicated that the structure of carbon nanotubes was not changed after oxidation.

### 3.2. The Effects of oMWCNTs on Inducing Cd-MT

**Figure 3:**
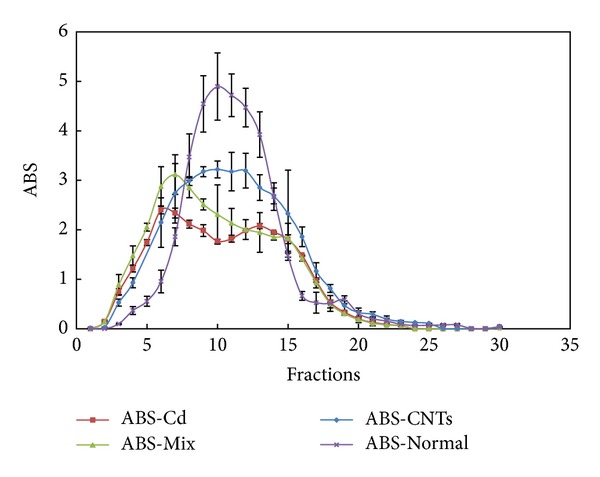
The eluent curves of processing liquid from liver tissues homogenate (ABS is the abbreviation of absorbance, absorption wavelength is 255 nm for ABS-Cd and ABS-Mix, and 260 nm for ABS-CNTs and ABS-Normal; *n* = 4 mice).

It was reported that the UV adsorption peak of Cd-MT was about 250 nm [28], the maximum absorption was 255 nm for eluent of accumulated Cd-MT mice liver in this work, and two peaks in the curve of ABS-fractions could be observed, which was similar to the results of exposure group to oMWCNTs (Figure 3), but the second peak was lower than the first elution peak and the detailed reasons would be discussed in the following section.

**Figure 4:**
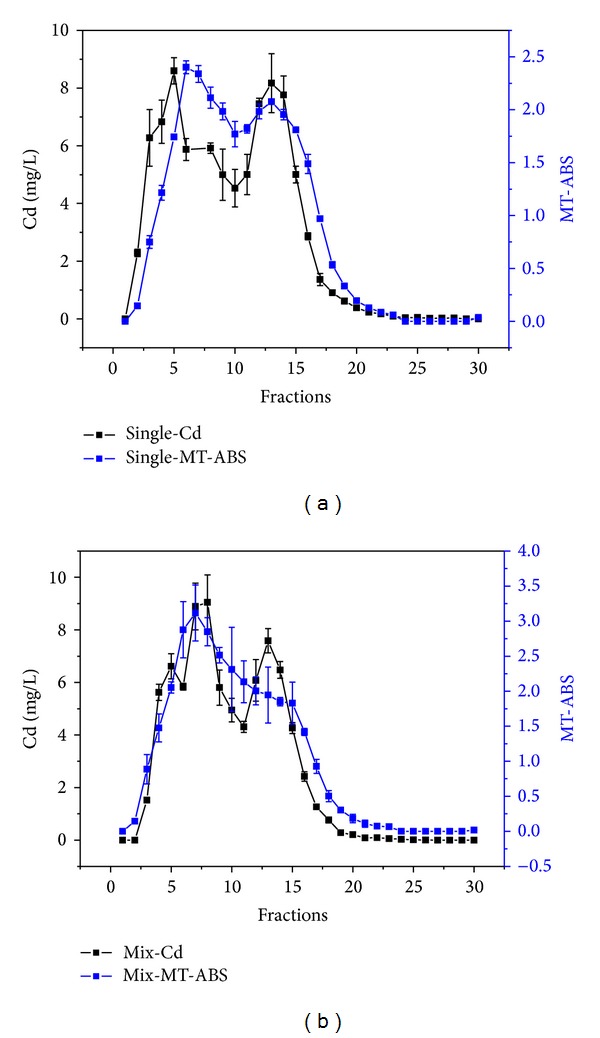
The changing curves of Cd concentration in Cd-MT are consistent with the absorption curves of MT (ABS is the abbreviation of absorbance; (a) is for Cd-MT model mice group; (b) is for coexposure mice group; *n* = 4 mice).

However, the maximum absorption in elution was 260 nm for normal and single exposure groups (Figure 3) and there was only one peak in ABS-fractions curve. The changes of Cd content in elution of Cd-MT model and coexposure groups were consistent with Cd-MT elution curve (Figure 4). The results indicated that Cd could induce MT synthesis and accumulate in liver in the form of Cd-MT, but single oMWCNTs exposure could not induce MT synthesis. In addition, after exposure of oMWCNTs to Cd-MT model mice, oMWCNTs could affect the synthesis of Cd-MT.

### 3.3. The Effects of oMWCNTs on Cd Hepatotoxicity

**Figure 5:**
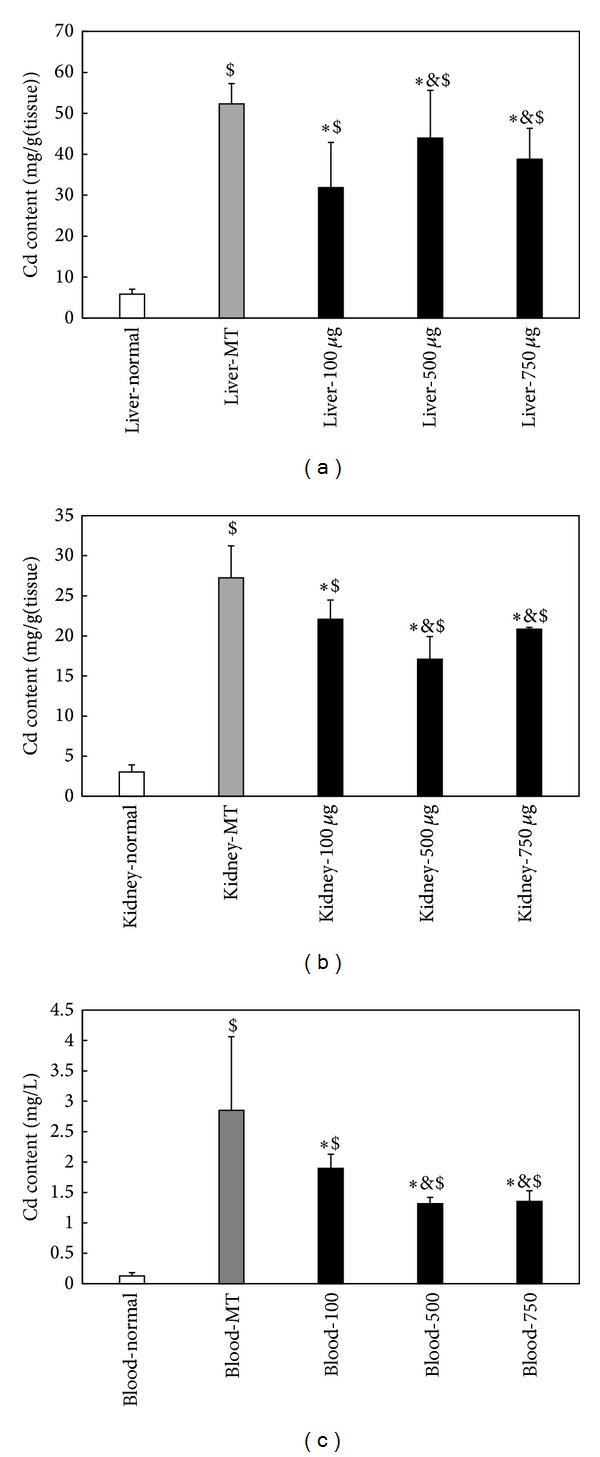
Release of Cd^2+^ from Cd-MT after exposure of different dosages of oMWCNTs to Cd-MT model mice. ((a) For Cd from liver, (b) for Cd from kidney, and (c) for Cd from blood; *P < 0.05, groups versus MT group; ^$^ *P* < 0.05, groups versus normal group; ^&^ *P* < 0.05, groups versus K-100 *μ*g. *n* = 6 mice).

The results of Figure 5 showed the distribution of Cd content in liver, kidney, and blood after exposure of different doses of oMWCNTs to Cd-MT model mice. Compared with the Cd-MT model group, the Cd contents were significantly lower in liver, kidney, and blood of exposure groups (Figure 5(a), ∗*P* < 0.05), indicating that oMWCNTs could release Cd from Cd-MT in tissues; the Cd content of exposure group to 100 *μ*g/mouse oMWCNTs was less than that of 500 and 750 *μ*g/mouse for liver tissue, but higher than that for kidney and blood, which showed that the low-dosage of oMWCNTs had a stronger influence on Cd-MT* in vivo*. Because free Cd was absorbed onto oMWCNTs and agglomerated in organism together with oMWCNTs, the influence of exposure group to 750 *μ*g/mouse oMWCNTs was obviously higher than that to 500 *μ*g/mouse.

**Figure 6:**
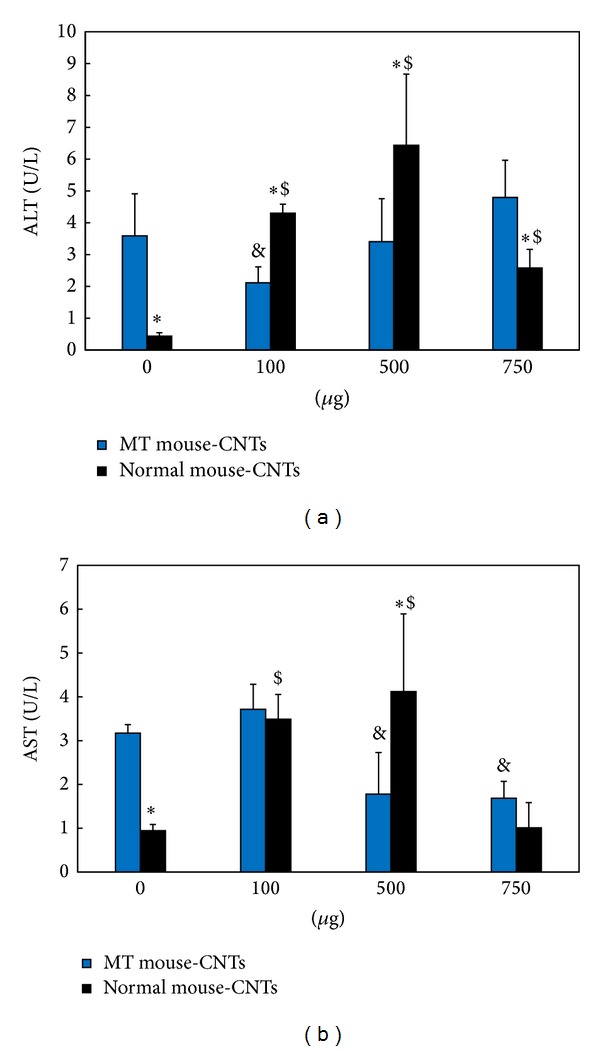
The effects of oMWCNTs on Cd hepatotoxicity and renal toxicity. ((a) For the changing of alanine aminotransferase (ALT) and (b) for the changing of aspartate aminotransferase (AST); *P < 0.05 for Normal-CNTs versus MT-CNTs at the same CNTs dose; ^&^ *P* < 0.05 for groups versus MT-0 *μ*g CNTs group; ^$^ *P* < 0.05 for groups versus Normal-0 *μ*g CNTs Groups; *n* = 6 mice).

**Figure 7:**
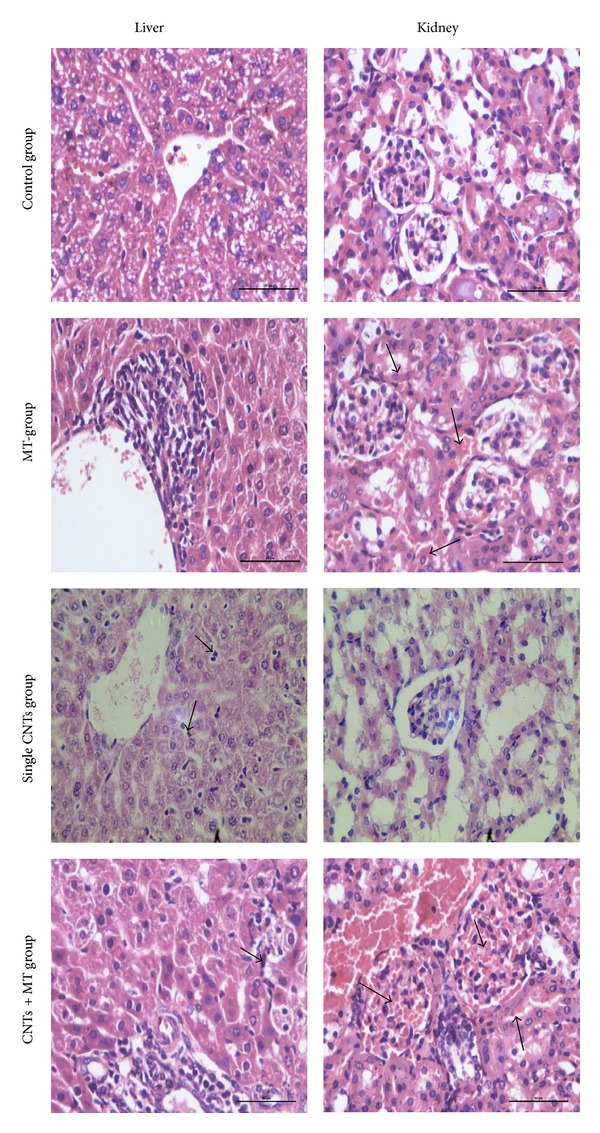
The histology of liver and kidney of control, MT, single CNTs exposure, and coexposure groups. (The arrows in the figure showed the pathological changes of tissue.)

**Figure 8:**
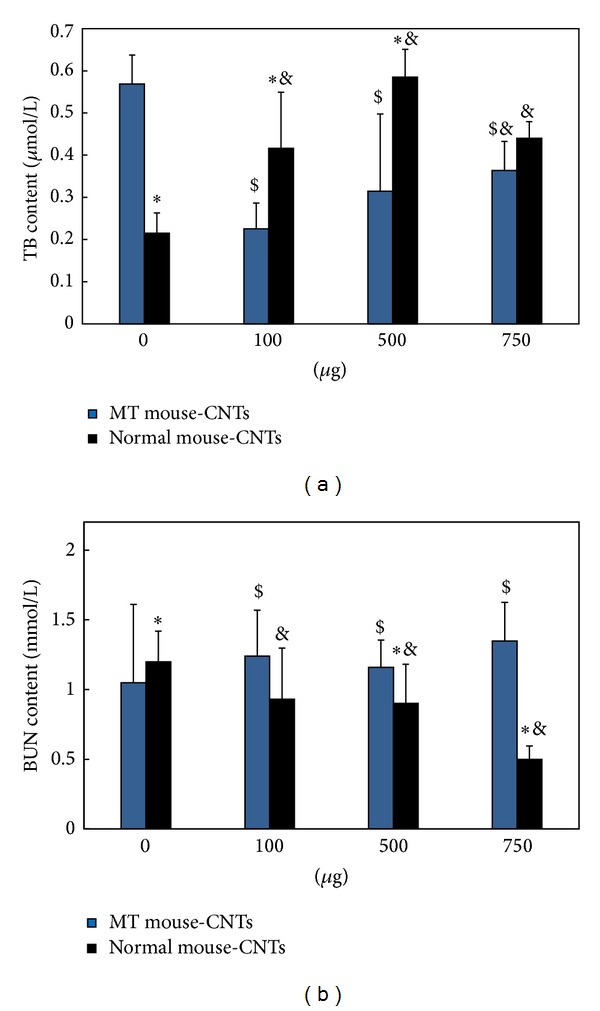
The effects of oMWCNTs on Cd renal toxicity. ((a) For the changing of total bilirubin (TB) and (b) for the changing of blood urea nitrogen (BUN); *P < 0.05 for Normal-CNTs versus MT-CNTs at the same CNTs dose; ^&^ *P* < 0.05 for groups versus Normal-0 *μ*g CNTs group; ^$^ *P* < 0.05 for groups versus MT-0 *μ*g CNTs groups; *n* = 6 mice).

The ALT content in blood plasma decreased after exposing different doses of oMWCNTs to Cd-MT model mice (Figure 6(a)), but the decreased tendency was weakening with the increasing of oMWCNTs dose. For the exposure group with 750 *μ*g/mouse oMWCNTs, the ALT content showed no difference with the Cd-MT model group. Compared to the single oMWCNTs exposure group, the ALT content of the coexposure group was significantly lower (Figure 6(a)). For the 750 *μ*g/mouse exposure groups, the ALT levels of single exposure group were lower than that of coexposure group. As shown in Figure 6(b), when the exposure concentration reached 500 *μ*g/mouse or 750 *μ*g/mouse for Cd-MT model mice, the AST levels in blood plasma began to decrease. However, compared with the single exposure groups, the AST content of the coexposure groups showed no significant difference, indicating that the impact of oMWCNTs on the secretion of AST was poor for Cd-MT model mice. As the histology of kidney and liver of all groups shown (Figure 7), compared with the control group, the histology indicated that the kidney tissue of MT group and the liver tissue of single oMWCNTs exposure group were minimal lesion disease, but the kidney of coexposure group showed serious pathological changes, including tissue edema, bleeding, and the karyotheca dissolved. And the further investigation also showed that the TB content of coexposure group to 100 *μ*g/mice or 500 *μ*g/mice oMWCNTs was lower than that of single exposure to Cd or oMWCNTs (Figure 8(a)); and after single exposure to normal mice with different oMWCNTs doses, the BUN content in plasma would be less than that in MT mice, which might reflect the disorder of kidney, but the BUN level was almost close to normal level (Figure 8(b)). And those results were according to the result of histology.

## 4. Discussion

The present work studied preliminarily the behavior and hepatotoxicity of heavy metal ions (Cd^2+^) in presence of oMWCNTs in mice. It was proposed that the maximum absorption peak appeared around 250 nm for Cd-MT [28] and the results of the experiment illustrated that oMWCNTs could not induce the liver of mice synthesis MT, and Cd^2+^ induced successfully Cd-MT synthesis in liver tissue in mice (Figures 3 and 4). Yanjiao et al. reported that MT folded into two separate domains (α and *β*) exhibiting different structures and functions independently, and Cd preferred to bind with α domains compared to *β* domains. In addition, the absorption peak for *β* domains at 254 nm was less significant than that for α domains [29]. This may be the reasons that the elution curve shows two different strength peaks for the Cd-MT model and coexposure groups (Figure 4(b)).

Our previous works indicated that oMWCNTs accumulated mainly in liver, lung, spleen, and kidney in mice after injection intravenously [15]; and Cd^2+^ accumulated in liver and kidney in the form of Cd-MT* in vivo *[10]. The authors chose the changes of Cd^2+^ content in blood, liver, and kidney to investigate the influence of oMWCNTs on Cd-MT in mice. After oMWCNTs exposure to Cd-MT model mice, oMWCNTs interacted with the accumulated Cd-MT in liver and kidney and then released Cd^2+^ from Cd-MT into blood circulatory system (Figure 5). Most of free Cd^2+^ would be eliminated through circulatory system, leading to the decrease of Cd^2+^ in blood, liver, and kidney and increase of toxicity of Cd^2+^ in kidney, which could be seen from the histology of the kidney and liver tissue (Figure 7) and the level of BUN and TB; but another part of Cd^2+^ adsorbed onto oMWCNTs continued to accumulate in tissues together with oMWCNTs. Because the content of oMWCNTs in liver increased with the increase of injection dosage of oMWCNTs, more free Cd^2+^ ions retained together with oMWCNTs in the liver, resulting in the decrease of Cd^2+^ content in the kidney (Figure 5). However, when the Cd-MT model mice were exposed to 750 *μ*g/mouse oMWCNTs, oMWCNTs were intercepted in lungs for the aggregation of high concentrations of oMWCNTs [30], and less oMWCNTs entered into liver and kidney; thus the effect on Cd-MT was reduced (Figure 5).

It was reported that oMWCNTs would be retained for long time in liver, lung, spleen, and other tissues after entering into body and damaged the tissues through inducing inflammation, granuloma, cell apoptosis and DNA damage, and so on [18, 20]. Cd^2+^ stimulated the liver and kidney to produce MT after exposure and was accumulated in the form of Cd-MT in tissues. After oMWCNTs exposure to Cd-MT model mice, oMWCNTs could release Cd^2+^ from Cd-MT in liver into circulation system, and then Cd^2+^ was eliminated out from body to decrease its accumulation in liver. The Cd^2+^ hepatotoxicity was decreased for liver (Figures 5 and 6), but the released Cd^2+^ ions could enter into kidney and increase their damage to kidney (Figures 7 and 8). Some researchers also found that some proteins would combine with oMWCNTs* in vivo*, which could improve the biological compatibility and metabolic capacity of oMWCNTs and the biological toxicity of oMWCNTs was reduced [31]. Therefore, authors thought that MT as a kind of protein released from Cd-MT could bind with oMWCNTs to decrease the hepatotoxicity of oMWCNTs, leading to the fact that the content of ALT/AST/TB in plasma of Cd-MT model mice group was lower than that of single exposure to normal mice group with different oMWCNTs doses (Figures 6 and 8(a)). The detailed mechanism needs to be further investigated. Because the Cd^2+^ could be released from Cd-MT and the MT combined with oMWCNTs, the coexposure of Cd^2+^ and oMWCNTs to mice could decrease the hepatotoxicity significantly compared to single exposure. However, when the coexposure dosage of oMWCNTs was 750 *μ*g/mouse, most parts of oMWCNTs were retained in the lung tissue, which could reduce their damage to liver, and so their negative effects were less than those of low doses (100 *μ*g/mice or 500 *μ*g/mice) exposure groups.

## 5. Conclusion 

The model mice with Cd-MT were made via injection with Cd^2+^ and then they were exposed to different dosages of oMWCNTs. The results showed that oMWCNTs could cause Cd^2+^ releasing from the accumulated Cd-MT. After the oMWCNTs and Cd^2+^ were injected together or respectively, the content changes of ALT, AST, TB, and BUN in plasma and the histology of liver and kidney both indicated that the hepatotoxicity of coexposure was lower than that of single exposure. It can be deduced that MT could be connected with oMWCNTs to reduce their hepatotoxicity, but the detailed mechanisms needed further study.

## Supplementary Material

In sum, oMWCNTs could cause Cd^2*+*^ release from the accumulated Cd-MT; in addition, after the oMWCNTs and Cd injected together or respectively, the content changes of hormone in plasma and liver tissues indicated that the hepatotoxicity of co-exposure was lower than that of single exposure. The detailed flow chart was provided in Supplementary Material.
