# Supplementary material for: The Effect of Multiwalled Carbon Nanotubes on Hepatotoxicity of Cd2+ in Accumulated Cadmium-Metallothione in Mice
Source: Biomed Res Int. 2014 Sep 2;2014:463161. doi: 10.1155/2014/463161 (PMC4167650; doi:10.1155/2014/463161)

Graphical Abstract

The model mice with Cd-MT were made via injection with Cd^{2+}, and then they were exposed to different dosages of oMWCNTs, the results showed that oMWCNTs could cause Cd2+ release from the accumulated Cd-MT; after the oMWCNTs and Cd injected together or respectively, the content changes of ALT and AST in plasma indicated that the hepatotoxicity of co-exposure was lower than that of single exposure, therefore, the author inferred that MT could be connected with oMWCNTs to reduce their hepatotoxicity.

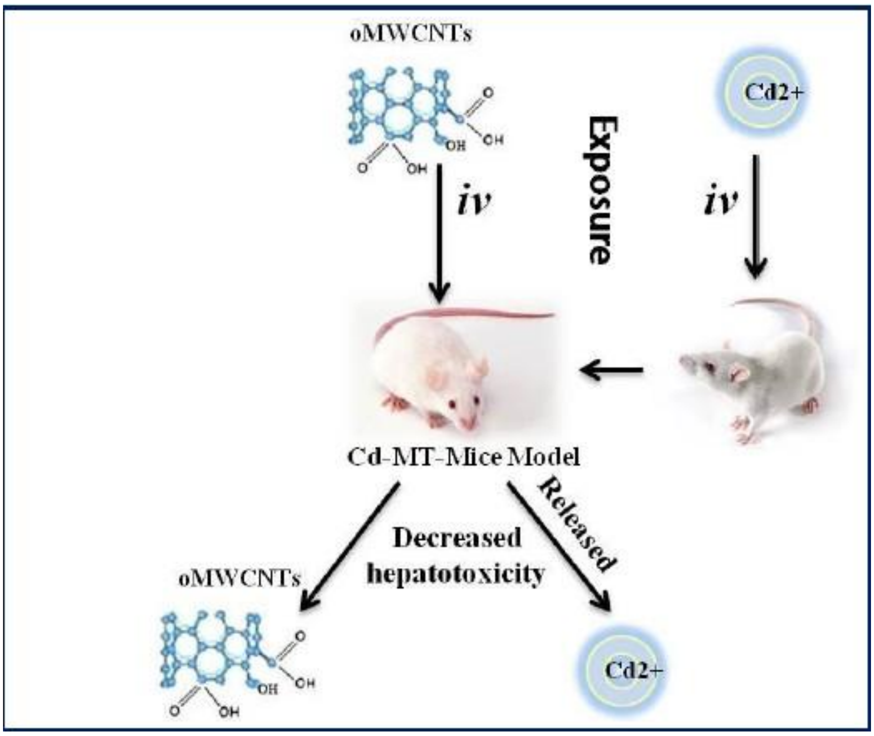

Supplement: Supplementary file 1 — In sum, oMWCNTs could cause Cd2+ release from the accumulated Cd-MT; in addition, after the oMWCNTs and Cd injected together or respectively, the content changes of hormone in plasma and liver tissues indicated that the hepatotoxicity of co-exposure was lower than that of single exposure. The detailed flow chart was provided in Supplementary Material. [file 463161.f1.pdf]
